# Increased intrinsic and synaptic excitability of hypothalamic POMC neurons underlies chronic stress-induced behavioral deficits

**DOI:** 10.1038/s41380-022-01872-5

**Published:** 2022-12-06

**Authors:** Xing Fang, Yuting Chen, Jiangong Wang, Ziliang Zhang, Yu Bai, Kirstyn Denney, Lin Gan, Ming Guo, Neal L. Weintraub, Yun Lei, Xin-Yun Lu

**Affiliations:** 1 Department of Neuroscience & Regenerative Medicine, Medical College of Georgia at Augusta University, Augusta, GA USA; 2 Department of Medicine, Vascular Biology Center, Medical College of Georgia at Augusta University, Augusta, GA USA

**Keywords:** Neuroscience, Depression

## Abstract

Chronic stress exposure induces maladaptive behavioral responses and increases susceptibility to neuropsychiatric conditions. However, specific neuronal populations and circuits that are highly sensitive to stress and trigger maladaptive behavioral responses remain to be identified. Here we investigate the patterns of spontaneous activity of proopiomelanocortin (POMC) neurons in the arcuate nucleus (ARC) of the hypothalamus following exposure to chronic unpredictable stress (CUS) for 10 days, a stress paradigm used to induce behavioral deficits such as anhedonia and behavioral despair [1, 2]. CUS exposure increased spontaneous firing of POMC neurons in both male and female mice, attributable to reduced GABA-mediated synaptic inhibition and increased intrinsic neuronal excitability. While acute activation of POMC neurons failed to induce behavioral changes in non-stressed mice of both sexes, subacute (3 days) and chronic (10 days) repeated activation of POMC neurons was sufficient to induce anhedonia and behavioral despair in males but not females under non-stress conditions. Acute activation of POMC neurons promoted susceptibility to subthreshold unpredictable stress in both male and female mice. Conversely, acute inhibition of POMC neurons was sufficient to reverse CUS-induced anhedonia and behavioral despair in both sexes. Collectively, these results indicate that chronic stress induces both synaptic and intrinsic plasticity of POMC neurons, leading to neuronal hyperactivity. Our findings suggest that POMC neuron dysfunction drives chronic stress-related behavioral deficits.

## Introduction

Chronic stress induces maladaptive behaviors and triggers the development of neuropsychiatric disorders, including depression, anxiety, and cognitive dysfunction. Extensive studies have focused on the brain regions that are typically associated with emotional, motivational and cognitive processes, such as the prefrontal cortex, hippocampus and amygdala, in these stress-related disorders [3]. However, the neural substrates and the precise circuit mechanisms that drive maladaptive behaviors and contribute to vulnerability to neuropsychiatric conditions remain poorly understood. The arcuate nucleus (ARC), located in the mediobasal hypothalamus around the third ventricle near the median eminence, has emerged as a brain site integrating and coordinating neural, neuroendocrine and behavioral responses to stress [1, 4–12].

The ARC contains two distinct populations of neurons that express proopiomelanocortin (POMC) or agouti-related protein (AgRP). POMC-derived alpha-melanocyte-stimulating hormone (α-MSH) is an endogenous agonist that activates melanocortin 3 and 4 receptors, whereas AgRP acts as an endogenous antagonist at the same receptors [13]. POMC and AgRP neurons in the ARC exhibit similar projection patterns throughout the brain [6, 14], innervating brain regions involved in neuroendocrine control and adaptive behaviors related to stress, such as the paraventricular nucleus of the hypothalamus (PVN), bed nucleus of the stria terminalis, and amygdala [14]. Nonetheless, these two distinct neuronal populations have so far predominately been studied in the context of feeding and energy balance [15–23]. However, while stimulating AgRP neurons induces a rapid and robust feeding response and weight gain, activation of POMC neurons causes only a marginal effect on feeding and body weight [17, 24, 25], which is in contrast to pharmacological studies with melanocortin receptor agonists [4, 5, 26]. We and others have demonstrated that central injection of α-MSH or its analogs induces stress-like endocrine and behavioral reactions [5, 27, 28], whereas blockade of melanocortin 4 receptors attenuates endocrine and behavioral responses to stress [27, 29–31]. Importantly, POMC gene variants in humans have been reported to interact with stress life events and associate with antidepressant treatment responses [32]. Exposure to different types of stressors such as restraint, immobilization or inescapable foot shock increases expression levels of POMC mRNA in the ARC [33–35]. We have previously shown that POMC neurons in the ARC can be activated rapidly by acute restraint and forced swim stress [4]. Likewise, POMC neurons recorded after acute stress or in the acute phase after repeated stress exposure exhibit hyperexcitability [9]. These results suggest that the endogenous POMC system is involved in stress responses.

Recently, we have shown that chronic unpredictable stress (CUS), a stress paradigm that generates behavioral deficits such as anhedonia and behavioral despair [1, 2], suppresses AgRP neuron activity through increasing synaptic inhibition and decreasing intrinsic neuronal excitability [1]. This hypoactivity of AgRP neurons correlates with the expression of CUS-induced behavioral deficits [1]. Moreover, direct stimulation of AgRP neurons was sufficient to reverse CUS-induced anhedonia and behavioral despair [1]. Given the anatomical and functional interactions with AgRP neurons, we hypothesize that POMC neurons may also undergo chronic stress-induced synaptic and intrinsic plasticity to modulate behavioral adaptation. In this study, we set out to determine how POMC neurons undergo stress-induced plastic changes and contribute to shaping behavioral susceptibility to chronic stress. Several important questions were addressed: a) how chronic stress modulates excitatory and inhibitory synaptic transmission and intrinsic excitability of POMC neurons; b) whether stimulation of POMC neurons mimics stress-induced behavioral responses; and c) whether activation and inhibition of POMC neurons affect stress susceptibility and chronic stress-induced behavioral deficits. To answer these questions, two lines of transgenic reporter mice were used for whole-cell patch clamp recordings to determine synaptic inputs and intrinsic membrane properties of POMC neurons following stress exposure. Additionally, a Cre-dependent DREADDs (Designer Receptors Exclusively Activated by Designer Drugs) approach was employed to remotely manipulate POMC neuron activity to test the causal relationship between POMC neuron activity and behavioral consequences.

## Materials and methods

### Animals

Wild-type C57BL/6J, *Pomc-Cre* mice (Stock No. 005965), *Pomc-GFP* mice (Stock No. 009593) and *Ai14* mice (Stock No. 007914) were purchased from Jackson Laboratory (Bar Harbor, ME, USA). *Ai14* mice have a loxP-flanked STOP cassette preventing transcription of a CAG promoter-driven red fluorescent protein variant (tdTomato) and inserted into the *Gt(ROSA)26Sor* locus (Gt(ROSA)26Sor^tm14(CAG-tdTomato)^). *Ai14* mice express robust tdTomato fluorescence following Cre-mediated recombination [36]. Male *Pomc-Cre* mice were crossed with Ai14 tdTomato female mice to obtain *Pomc-Cre;tdTomato* mice with tdTomato fluorescence in Cre-expressing cells, which was used to identify POMC neurons. All animal procedures were approved by the Institutional Animal Care and Use Committees of University of Texas Health Science Center at San Antonio and Augusta University. For further details see SI Materials and Methods.

### Viral injections

*Pomc-Cre* mice at 7 weeks of age were used for virus injection as described elsewhere [1, 2, 37]. For further details see SI Materials and Methods.

### Whole-cell patch-clamp recordings

Electrophysiological recordings were performed as previously described [1, 2, 38]. For further details see SI Materials and Methods.

### Behavioral procedures

Behavioral tests were performed in adult female and male mice at 9–11 weeks of age. Animals were transferred to a testing room and habituated to the room conditions for 3–4 h before the beginning of behavioral experiments. Behavioral testing procedures were performed in the late light cycle except for the sucrose preference test, which was carried out during the first 2 h of the dark cycle. For the behavioral tests involving chemogenetic activation or inhibition, mice received an intraperitoneal (i.p.) injection of 0.3 mg/kg clozapine N-oxide (CNO; Sigma-Aldrich, Saint Louis, MO, USA) 30 min before testing. Behaviors were scored by investigators who were blinded to the treatments.

#### Chronic unpredictable stress

Mice (7–9 weeks old) were subjected to different types of stressors at different times of the day for 10 consecutive days. The stressors included 2-h restraint, 15-min tail pinch, 24-h constant light, 24-h wet bedding with 45° cage tilt, 10-min inescapable foot shocks, 30-min elevated platform and social isolation (Table 1). Stress procedures were conducted in a procedure room. Mice exposed to the CUS procedure were singly housed. Control mice were group housed and briefly handled daily in the housing room.

**Table 1:** Subthreshold and chronic unpredictable stress procedures.

	Stressors			
	AM	PM		
Day 1	2-h restraint	15-min tail pinch	SUS^a^ (3 days)	CUS^a^ (10 days)
Day 2	24-h constant light	2-h restraint		
Day 3	2-h restraint	24-h 45° cage tilt and wet bedding		
Day 4	10-min inescapable shock (0.3 mA, 2-s duration, at random intervals with an average of 16 s)	2-h restraint		
Day 5	2-h restraint	30-min elevated platform		
Day 6	15-min tail pinch	2-h restraint		
Day 7	2-h restraint	24-h constant light		
Day 8	24-h 45° cage tilt and wet bedding	2-h restraint		
Day 9	2-h restraint	10-min inescapable shock (0.3 mA, 2-s duration, at random intervals with an average of 16 s)		
Day 10	30-min elevated platform	2-h restraint		

^a^*SUS* subthreshold unpredictable stress, *CUS* chronic unpredictable stress.

For further details of each behavioral test, see SI Materials and Methods.

### Statistical analysis

All results are presented as mean ± s.e.m. (standard error of mean). Statistical analyses were performed using GraphPad Prism 8.0 (GraphPad Software, Inc., CA). The Shapiro–Wilk test and the F test were used to test the normality and the equality of variances, respectively. For further details of statistical analysis, see SI Materials and Methods.

## Results

### Chronic unpredictable stress alters spontaneous firing patterns of POMC neurons

**Fig. 1:**
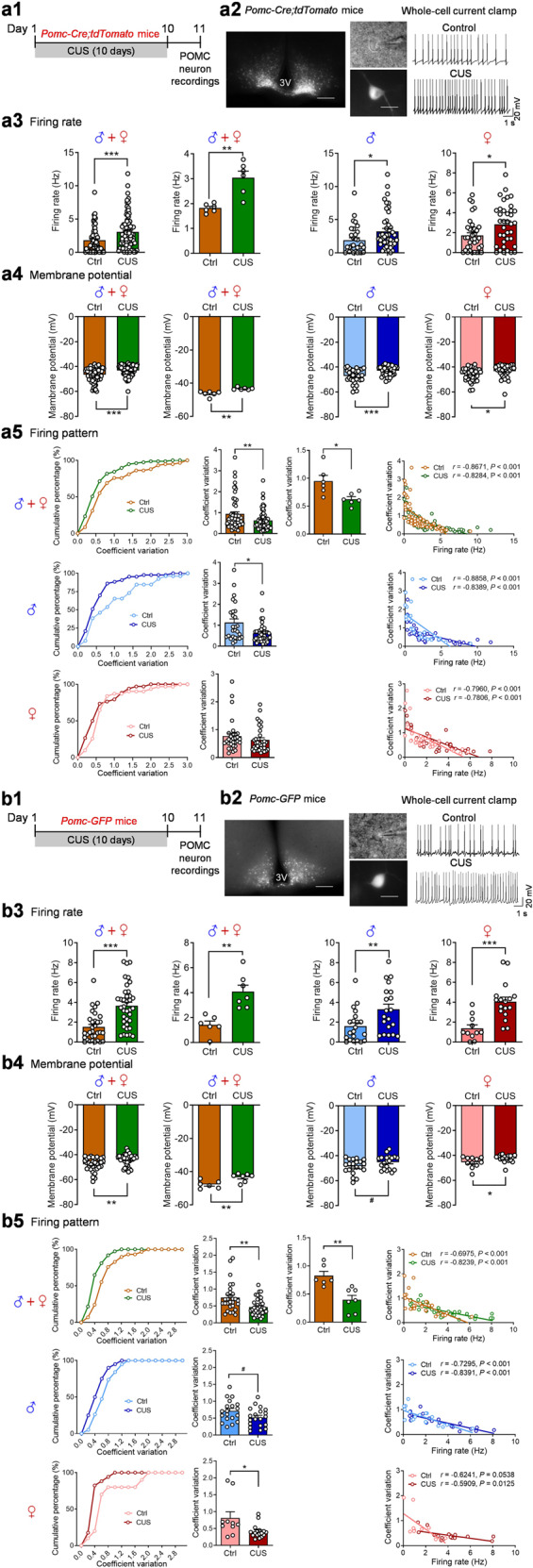
Chronic unpredictable stress modulates spontaneous firing patterns of POMC neurons. *Pomc-Cre;tdTomato* mice. **a1** Timeline of experimental procedures. **a2** Left, representative fluorescent images of a coronal brain slice from a *Pomc-Cre;tdTomato* mouse showing fluorescent POMC neurons in the arcuate nucleus (ARC). Scale bars, 200 µm for low magnification (5×) and 20 µm for high magnification (40×). Right, representative traces of spontaneous action potentials of POMC neurons from control and CUS groups. **a3**, **a4** Spontaneous firing rate (a3) and membrane potential (a4). Left, male and female combined, individual neurons (firing rate: Mann-Whitney test, *P* < 0.001; membrane potential: Mann-Whitney test, *P* < 0.001); middle-left, male and female combined, group neurons per mouse (firing rate: Welch’s test, *P* = 0.0049; membrane potential: Mann-Whitney test, *P* = 0.0022); middle-right, male mice-individual neurons (firing rate: Mann Whitney test, *P* = 0.0129; membrane potential: Mann Whitney test, *P* < 0.001); right, female mice-individual neurons (firing rate: Mann Whitney test, *P* = 0.0196; membrane potential: Mann Whitney test, *P* = 0.0256). **a5** Spontaneous firing patterns. Upper panel: spontaneous firing patterns from male and female mice combined. Left, cumulative probability distributions of coefficients of variation; middle-left, average coefficients of variation, individual neurons (Mann Whitney test, *P* = 0.0060); middle-right, average coefficients of variation, group neurons per mouse (*t*_(10)_ = 2.866; *P* = 0.0168); righ*t*, correlation analysis between spontaneous firing rates and coefficients of variation. Middle panel: spontaneous firing patterns from male mice (Mann Whitney test, *P* = 0.0102). Lower panel: spontaneous firing patterns from female mice (Mann Whitney test, *P* = 0.1266). Control (Ctrl): *n*  =  67 neurons from three male (31 neurons) and three female (36 neurons) mice. CUS: *n*  =  81 neurons from three male (44 neurons) and three female (37 neurons) mice. *Pomc-GFP* mice. **b1** Experimental timeline. **b2** Left, representative fluorescent images of a coronal brain slice from a *Pomc-GFP* mouse showing fluorescent POMC neurons in the ARC. Scale bars, 200 µm for low magnification (5×) and 20 µm for high magnification (40×). Right, representative traces of spontaneous action potentials of POMC neurons from control and CUS groups. **b3**, **b4** Spontaneous firing rate (b3) and membrane potential (b4). Left, male and female mice combined, individual neurons (firing rate: Mann Whitney test, *P* < 0.001; membrane potential: Mann Whitney test, *P* = 0.0027); middle-left, male and female mice combined, group neurons per mouse (firing rate: *t*_(11)_ = 4.244, *P* = 0.0014; membrane potential: *t*_(11)_ = 3.180, *P* = 0.0088); middle-right, male mice-individual neurons (firing rate: Mann-Whitney test, *P* = 0.0083; membrane potential: *t*_(39)_ = 1.973, ^#^*P* = 0.0556); right, female mice-individual neurons (firing rate: *t*_(26)_ = 4.228, *P* < 0.001; membrane potential: Mann Whitney test, *P* = 0.0215). **b5** Spontaneous firing patterns. Upper panel: male and female mice combined. Left, cumulative probability distributions of coefficients of variation; middle-left, average coefficients of variation, individual neurons (Mann Whitney test, *P* = 0.0016); middle-right, average coefficients of variation, group neurons per mouse (*t*_(11)_ = 3.867, *P* = 0.0026); right, correlation analysis between spontaneous firing rates and coefficients of variation. Middle panel: male mice (*t*_(37)_ = 2.011, ^#^*P* = 0.0517). Lower panel: female mice (Mann Whitney test, *P* = 0.0209). Ctrl: *n*  =  32 neurons from three male (21 neurons) and three female (11 neurons) mice. CUS: *n*  =  37 neurons from four male (20 neurons) and three female (17 neurons) mice. *P  <  0.05, **P  <  0.01, ***P  <  0.001 vs control group.

Our recent studies have demonstrated that repeated exposures to a variety of stressors in an unpredictable and uncontrollable manner for 10 consecutive days (CUS; Table 1) induce behavioral deficits in both male and female mice [1, 2]. Given the rapid responsiveness of POMC neurons in the ARC to acute stress [4], we examined whether chronic repeated exposure to stress causes persistent changes in the activity ARC POMC neurons. To visualize POMC neurons, *Pomc-Cre* mice were crossed with the *Ai14-tdTomato* mice to produce *Pomc-Cre;tdTomato* reporter mice, which enable the identification of tdTomato-positive cells as POMC neurons. Male and female *Pomc-Cre;tdTomato* mice at 7-8 weeks of age were subjected to 10 days of unpredictable stress, i.e., CUS. Whole-cell patch-clamp recordings under the current-clamp mode were made from POMC neurons of control mice and CUS mice 1 day after the last stress exposure (Fig. 1a1). First, data from male and female *Pomc-Cre;tdTomato* mice were combined for statistical analysis. We found that the frequency of spontaneous firing of POMC neurons was increased (Fig. 1a2–a3) and membrane potential was more depolarized after CUS exposure (Fig. 1a4). Moreover, we noticed that the percentage of silent POMC neurons (at frequencies <0.5 Hz) decreased by CUS [control 30% (20 out of 67 neurons); CUS 14% (11 out of 81 neurons)]. Then, male and female groups were analyzed separately to detect potential sex-specific effects of CUS. Both male and female mice exhibited increased spontaneous firing rates (Fig. 1a3) and depolarized membrane potential after CUS exposure (Fig. 1a4). These data indicate that POMC neurons become hyperactive after CUS exposure in mice of both sexes.

To determine the effects of CUS on firing patterns of POMC neurons, we analyzed the inter-spike interval distribution and the coefficient of variation, a measure of spike train irregularity. Under control conditions, POMC neurons displayed highly irregular spike times (coefficient of variation of the interspike intervals: male-mean, 1.127; female-mean, 0.7709). Analyses of the combined data from male and female mice revealed that CUS caused a shift in the cumulative probability distribution of interspike intervals to the left and resulted in a decrease in the coefficient of variation (Fig. 1a5). There was a negative correlation between firing rates and coefficients of variation (Fig. 1a5). These results indicate that POMC neurons fire more rapidly and regularly after CUS exposure. Further analysis of male and female groups separately showed that the cumulative probability distribution of interspike intervals was shifted to the left and the coefficient of variation was decreased by CUS in male but not in female mice (Fig. 1a5). Both male and female mice showed a negative correlation between firing rates and coefficients of variation under control and CUS conditions (Fig. 1a5).

In *Pomc-Cre;tdTomato* reporter mice, tdTomato-labeled POMC neurons could result from transient Cre expression during development [39]. To address this, we utilized *Pomc-GFP* mice to confirm the effects of CUS on the activity of POMC neurons. *Pomc-GFP* mice express enhanced green fluorescent protein (GFP) under control of the mouse *Pomc* promoter/enhancer regions, which accurately label the neurons with endogenous *Pomc* transcription in ARC [39, 40]. The stress procedure and the patch-clamp recording protocols used for *Pomc-GFP* mice were the same as used for *Pomc*-*Cre;tdTomato* mice (Fig. 1b1). First, data from male and female *Pomc-GFP* mice were combined for statistical analysis. Similar to that observed in *Pomc-Cre;tdTomato* mice, CUS resulted in an increase in firing rates (Fig. 1b2–b3) and a depolarization of the membrane potential (Fig. 1b4) of POMC neurons in mice of both sexes combined. Further analysis for male and female mice separately showed that CUS-induced changes in spontaneous firing of POMC neurons were not sex-specific. In addition, analysis of firing patterns of *Pomc-GFP* neurons revealed a shift in the cumulative frequency distribution of interspike intervals to the left and a decrease in the coefficient of variation of interspike intervals in male and female mice (Fig. 1b5). Additionally, a negative correlation between firing rates and coefficients of variation of interspike intervals was also confirmed in control and chronically stressed *Pomc-GFP* mice (Fig. 1b5). These data indicate that CUS increased firing rates and regularity of POMC neurons.

### Chronic unpredictable stress induces synaptic and intrinsic plasticity in POMC neurons

**Fig. 2:**
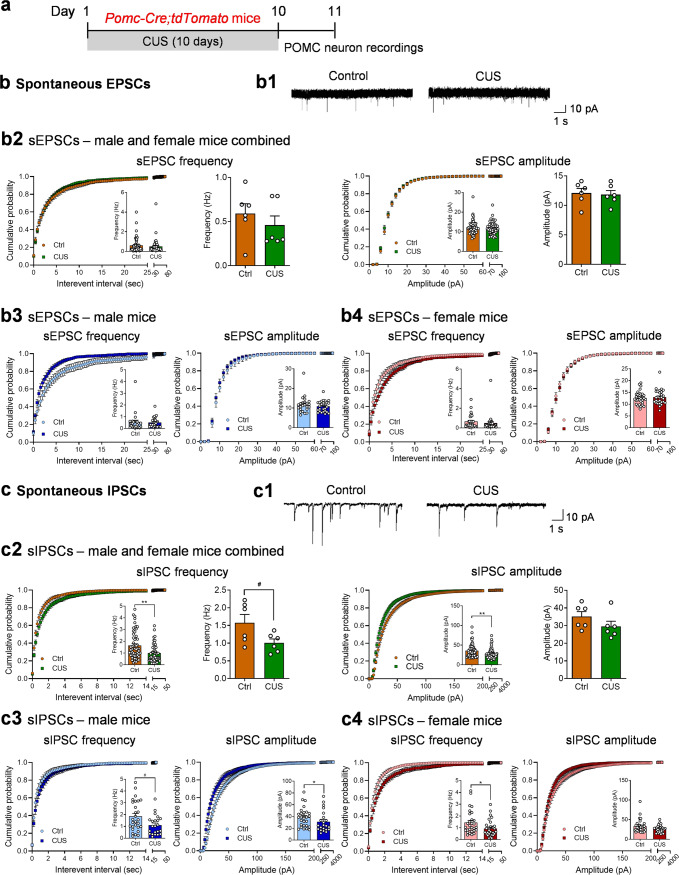

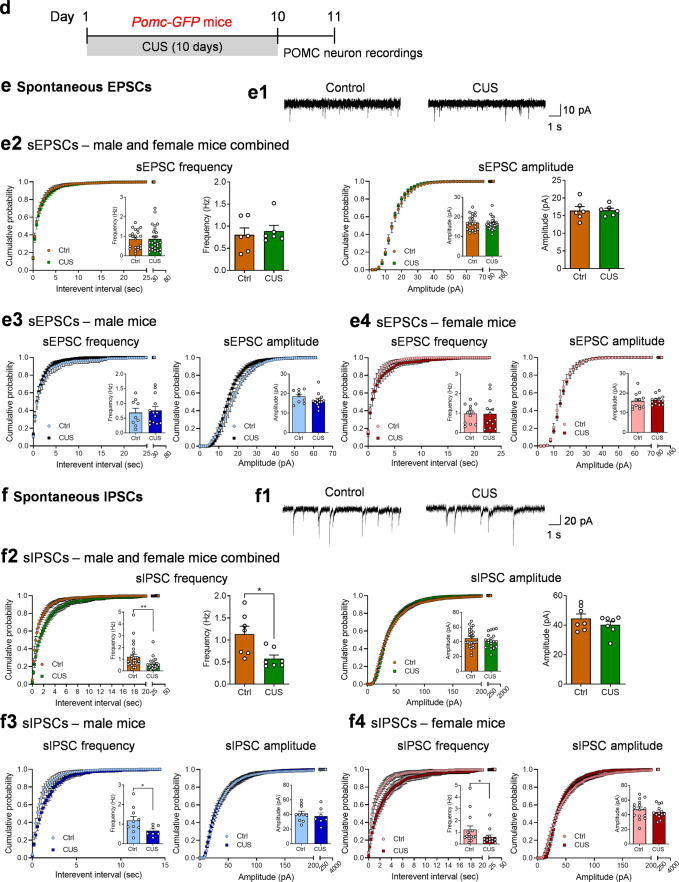

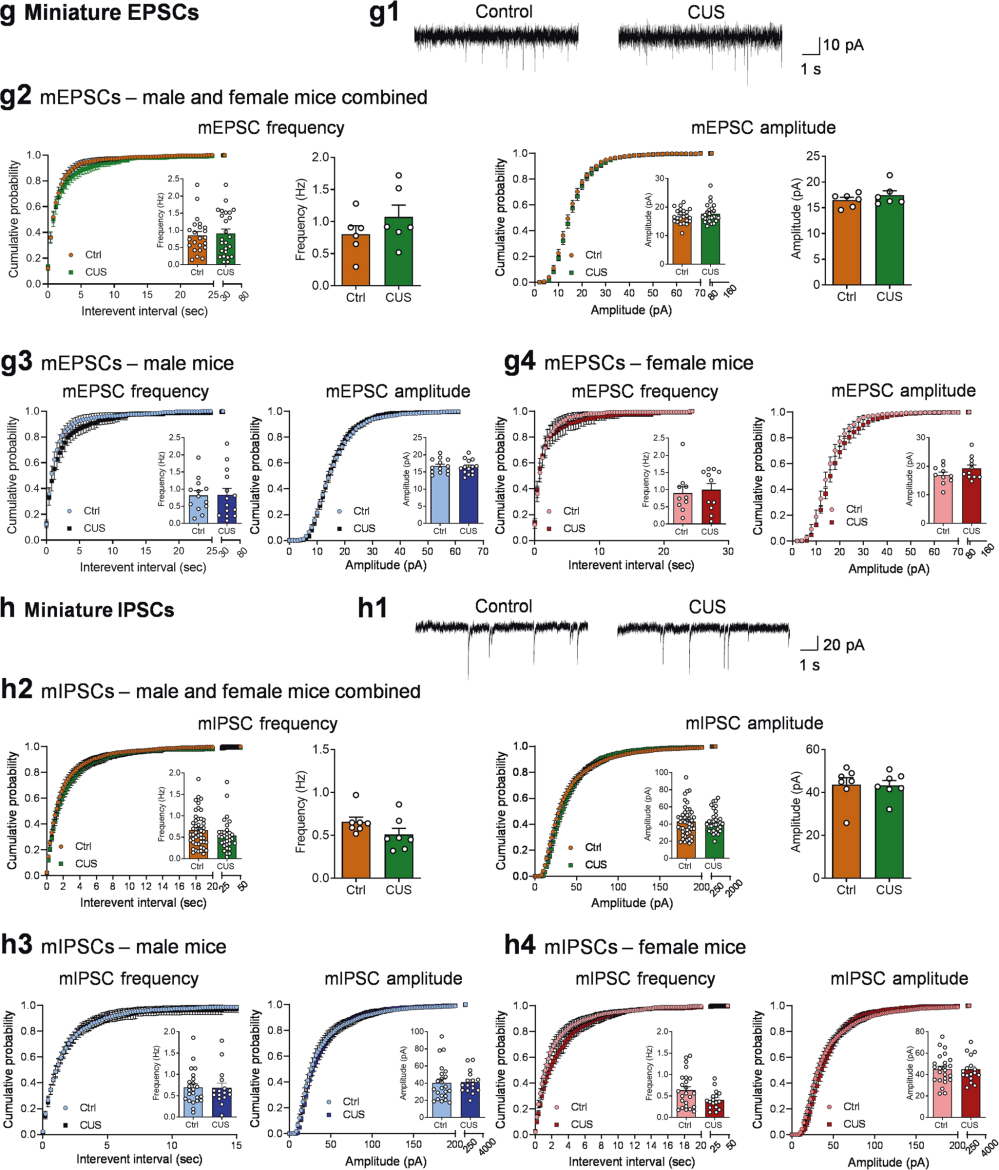
Chronic unpredictable stress affects spontaneous synaptic neurotransmission in POMC neurons. **a-c** Results from *Pomc-Cre;tdTomato* mice. **a** Experimental timeline. **b** Spontaneous EPSCs (sEPSCs) from *Pomc-Cre;tdTomato* mice. **b1** Representative traces depicting sEPSCs. **b2** sEPSCs from male and female *Pomc-Cre;tdTomato* mice combined. Left, cumulative probability plot for the interevent interval. Left insert, average frequency of sEPSCs, individual neurons (Mann Whitney test, *P* = 0.5796). Middle-left, average frequency of sEPSCs, group neurons per mouse (Mann Whitney test, *P* = 0.5887). Middle-right, cumulative probability plot for the amplitude. Middle-right insert, average amplitude of sEPSCs, individual neurons (Mann Whitney test, *P* = 0.6830). Right, average amplitude of sEPSCs, group neurons per mouse (*t*_(10)_ = 0.2574, *P* = 0.8021). **b3** sEPSC from male *Pomc-Cre;tdTomato* mice. Left, cumulative probability plot for the interevent interval. Left insert, average frequency of sEPSCs, individual neurons (Mann Whitney test, *P* = 0.3963). Right, cumulative probability plot for the amplitude. Right insert, average amplitude of sEPSCs, individual neurons (Mann Whitney test, *P* = 0.6875). **b4** sEPSC from female *Pomc-Cre;tdTomato* mice. Left, cumulative probability plot for the interevent interval. Left insert, average frequency of sEPSCs, individual neurons (Mann Whitney test, *P* = 0.1576). Right, cumulative probability plot for the amplitude. Right insert, average amplitude of sEPSCs, individual neurons (Mann Whitney test, *P* = 0.8833). Ctrl: *n* = 55 neurons from three male (24 neurons) and three female (31 neurons) mice. CUS: *n*  = 56 neurons from three male (27 neurons) and three female (29 neurons) mice. **c** Spontaneous IPSCs (sIPSCs) from *Pomc-Cre;tdTomato* mice. **c1** Representative traces depicting sIPSCs. **c2** sIPSC from male and female *Pomc-Cre;tdTomato* mice combined. Left, cumulative probability plot for the interevent interval. Left insert, average frequency of sIPSCs, individual neurons (Mann Whitney test, *P* = 0.0016). Middle-left, average frequency of sIPSCs, group neurons per mouse (*t*_(10)_ = 2.190, ^#^*P* = 0.0534). Middle-right, cumulative probability plot for the amplitude. Middle-right insert, average amplitude of sIPSCs, individual neurons (Mann Whitney test, *P* = 0.0069). Right, average amplitude of sIPSCs, group neurons per mouse (*t*_(10)_ = 1.390, *P* = 0.1948). **c3** sIPSC from male *Pomc-Cre;tdTomato* mice. Left, cumulative probability plot for the interevent interval. Left insert, average frequency of sIPSCs, individual neurons (Mann Whitney test, ^#^*P* = 0.0588). Right, cumulative probability plot for the amplitude. Right insert, average amplitude of sIPSCs, individual neurons (Mann Whitney test, *P* = 0.0466). **c4** sIPSC from female *Pomc-Cre;tdTomato* mice. Left, cumulative probability plot for the interevent interval. Left insert, average frequency of sIPSCs, individual neurons (Mann Whitney test, *P* = 0.0163). Right, cumulative probability plot for the amplitude (Mann Whitney test, *P* = 0.1321). Right insert, average amplitude of sIPSCs, individual neurons. Ctrl: *n* = 57 neurons from three male (28 neurons) and three female (29 neurons) mice. CUS: *n*  =  53 neurons from three male (22 neurons) and three female (31 neurons) mice. **d–h** Results from *Pomc-GFP* mice. **d** Experimental timeline. **e** sEPSCs from *Pomc-GFP* mice (**e1**-representative traces of sEPSCs in POMC neurons; **e2**-male and female combined: frequency-individual neurons, Mann Whitney test, *P* = 0.7626; frequency-group neurons per mouse, Mann Whitney test, *P* = 0.7294; amplitude-individual neurons: Mann Whitney test, *P* = 0.7265; amplitude-group neurons per mouse: *t*_(10)_ = 0.05081, *P* = 0.9605; **e3**-male only: frequency-individual neurons, Mann Whitney test, *P* = 0.7561; amplitude-individual neurons, *t*_(20)_ = 1.293, *P* = 0.2106; **e4**-female only: frequency-individual neurons, *t*_(23)_ = 0.03616, *P* = 0.9715; amplitude-individual neurons, *t*_(23)_ = 0.3747, *P* = 0.7113). Ctrl: *n*  =  21 neurons from three male (9 neurons) and three female (12 neurons) mice. CUS: *n*  =  26 neurons from three male (13 neurons) and three female (13 neurons) mice. **f** sIPSCs from *Pomc-GFP* mice (**f1**-representative traces of sIPSCs in POMC neurons; **f2**-male and female combined: frequency-individual neurons, Mann Whitney test, *P* = 0.0065; frequency-group neurons per mouse, Mann Whitney test, *P* = 0.0169; amplitude-individual neurons: *t*_(43)_ = 0.9380, *P* = 0.3535; amplitude-group neurons per mouse: Mann Whitney test, *P* = 0.6200; **f3**-male only: frequency-individual neurons, Unpaired *t* test with Welch’s correction, *P* = 0.0402; amplitude-individual neurons *t*_(15)_ = 0.6136, *P* = 0.5487; **f4**-female only: frequency-individual neurons, Mann Whitney test, *P* = 0.0401; amplitude-individual neurons, *t*_(26)_ = 0.8378, *P* = 0.4098). Ctrl: *n*  =  25 neurons from three male (10 neurons) and four female (15 neurons) mice. CUS: *n*  =  20 neurons from three male (7 neurons) and four female (13 neurons) mice. **g** Miniature EPSCs (mEPSCs) from *Pomc-GFP* mice (**g1**-representative traces of mEPSCs in POMC neurons; **g2**-male and female combined: frequency-individual neurons, Mann Whitney test, *P* = 0.9308; frequency-group neurons per mouse, *t*_(10)_ =  1.181, *P* = 0.2651; amplitude-individual neurons: Mann Whitney test, *P* = 0.4993; amplitude-group neurons per mouse: *t*_(10)_ = 0.9731, *P* = 0.3535; **g3**-male only: frequency-individual neurons, *t*_(25)_ = 0.07453, *P* = 0.9412; amplitude-individual neurons *t*_(25)_ = 0.3181, *P* = 0.7530; **g4**-female only: frequency-individual neurons, Mann Whitney test, *P* = 0.5930; amplitude-individual neurons, *t*_(19)_ = 1.484, *P* = 0.1543). Ctrl: *n* =  23 neurons from three male (13 neurons) and three female (10 neurons) mice. CUS: *n*  = 25 neurons from three male (14 neurons) and three female (11 neurons) mice. **h** Miniature IPSCs (mIPSCs) from *Pomc-GFP* mice (**h1**-representative traces of mIPSCs in POMC neurons; **h2**-male and female combined: frequency-individual neurons, Mann Whitney test, *P* = 0.1668; frequency-group neurons per mouse, Mann Whitney test, *P* = 0.1200; amplitude-individual neurons: Mann Whitney test, *P* = 0.7338; amplitude-group neurons per mouse: Mann Whitney test, *P* = 0.7104; **h3**-male only: frequency-individual neurons, Mann Whitney test, *P* = 0.9046; amplitude-individual neurons, Mann Whitney test, *P* = 0.4369; **h4**-female only: frequency-individual neurons, Mann Whitney test, *P* = 0.0943; amplitude-individual neurons, *t*_(39)_ = 0.05230, *P* = 0.9586). Ctrl: *n* = 47 neurons from three male (23 neurons) and four female (24 neurons) mice. CUS: *n*  = 33 neurons from three male (16 neurons) and three female (17 neurons) mice. *P < 0.05, **P < 0.01, ***P < 0.001 vs control group.

Alterations in synaptic drive could underlie the increased spontaneous firing rates in ARC POMC neurons. To test this possibility, we examined synaptic transmission at excitatory and inhibitory synapses of POMC neurons 1 day after the last stress exposure of CUS in *Pomc-Cre;tdTomato* mice (Fig. 2a). Whole-cell voltage-clamp recordings of EPSCs and IPSCs were performed at −60 mV holding potential. Spontaneous EPSCs, recorded in the presence of 100 μM picrotoxin, a GABA_A_ receptor antagonist used to block GABAergic transmission, in ARC POMC neurons showed no significant changes in the frequency or amplitude (Fig. 2b1–b4). Recordings of spontaneous IPSCs in POMC neurons were made in the presence of AMPA and NMDA receptor antagonists to block glutamatergic synaptic transmission (Fig. 2c1). CUS decreased the mean frequency and amplitude of spontaneous IPSCs when data were pooled from males and females (Fig. 2c2); similar trends were observed when data were analyzed separately by sex (Fig. 2c3, c4). These results suggest that both GABAergic drive to POMC neurons, a presynaptic effect, and POMC neuron responsiveness to GABA_A_ receptor activation, a postsynaptic response, were decreased by CUS, thus leading to synaptic disinhibition of POMC neurons.

The effects of CUS on spontaneous EPSCs and IPSCs in POMC neurons were also examined in the ARC of *Pomc-GFP* mice (Fig. 2d). Similar to the observations made in *Pomc-Cre;tdTomato* mice, neither the frequency nor the amplitude of spontaneous EPSCs was altered in POMC neurons from *Pomc-GFP* mice after CUS exposure (Fig. 2e). By contrast, CUS decreased the frequency, but not the amplitude, of spontaneous IPSCs in POMC neurons (Fig. 2f).

Spontaneous synaptic events (EPSCs and IPSCs) could be driven by action potential-dependent and/or -independent transmitter release. To determine whether CUS affects action potential-independent synaptic events, spontaneous, miniature EPSCs (mEPSCs) and miniature IPSCs (mIPSCs) were recorded in POMC neurons from *Pomc-GFP* mice in the presence of 1 µM tetrodotoxin to block sodium channels and action potentials. There were no significant changes in the frequency or amplitude of mEPSCs (Fig. 2g) or mIPSCs (Fig. 2h), suggesting that chronic stress facilitates synaptic inhibitory transmission through an action potential-dependent mechanism.

**Fig. 3:**
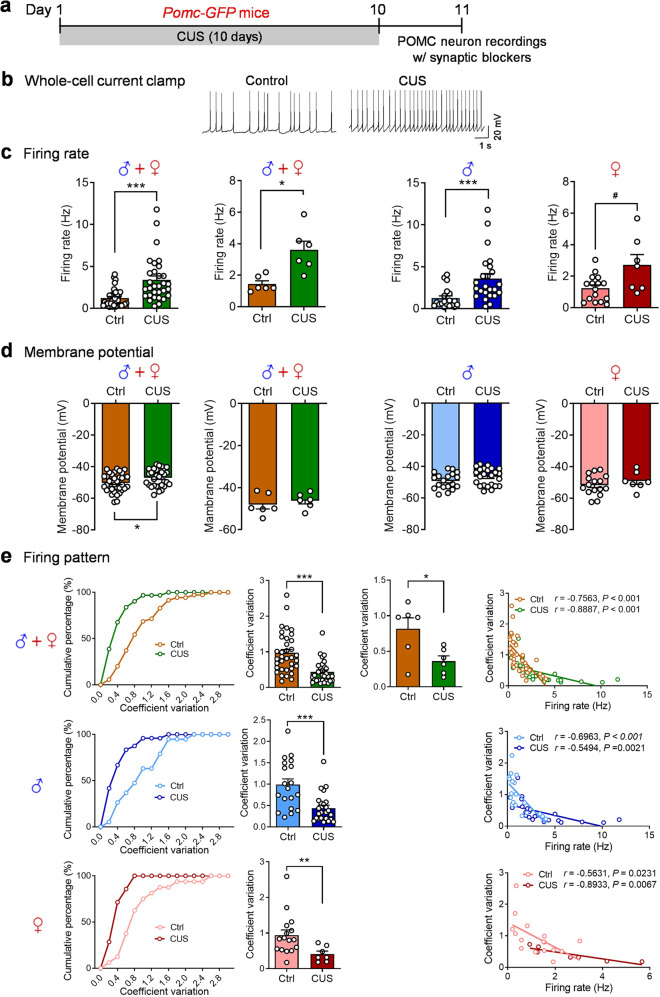

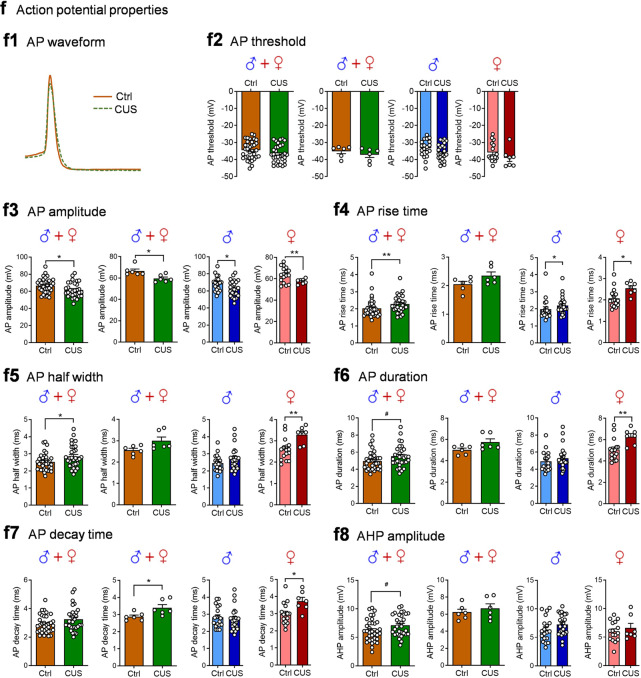
Chronic unpredictable stress increases intrinsic activity of POMC neurons. **a** Timeline of the CUS procedure and patch-clamp recordings of POMC neurons from *Pomc-GFP* mice in the presence of synaptic blockers. **b** Representative traces showing intrinsic action potentials of POMC neurons. Intrinsic firing rate (**c**) and membrane potential (**d**). Left, male and female mice combined, individual neurons (firing rate: Mann Whitney test, *P* < 0.001; membrane potential: *t*_(64)_ = 2.307, *P* = 0.0243); middle-left, male and female mice combined, group neurons per mouse (firing rate: unpaired t test with Welch’s correction, *P* = 0.0106; membrane potential: *t*_(10)_ = 0.7205, *P* = 0.4877); middle-right, male mice-individual neurons (firing rate: Mann Whitney test, *P* < 0.001; membrane potential: *t*_(41)_ = 1.630, *P* = 0.1108); right, female mice-individual neurons (firing rate: unpaired t test with Welch’s correction, ^#^*P* = 0.0685; membrane potential: *t*_(21)_ = 0.9288, *P* = 0.3636). **e** Intrinsic firing patt**e**rn. Upper panel: male and female mice combined. Left, cumulative probability distributions of coefficients of variation; middle-left, average coefficients of variation, individual neurons (Mann Whitney test, *P* < 0.001); middle-right, average coefficients of variation, group neurons per mouse (*t*_(10)_ = 2.669, *P* = 0.0235); righ*t*, correlation analysis between spontaneous firing rates and coefficients of variation. Middle panel: male mice (Mann Whitney test, *P* < 0.001). Lower panel: female mice (Mann Whitney test, *P* = 0.0076). **f** Action potential (AP) waveform. **f1** Representative AP waveforms recorded in POMC neurons from control (brown line) and CUS (green line) mice. **f2** AP threshold (male and female combined-individual neurons: *t*_(64)_ = 1.467, *P* = 0.1471; male and female combined-group neurons per mouse: *t*_(10)_ = 0.9658, *P* = 0.3569; male-individual neurons: *t*_(41)_ = 1.456, *P* = 0.1529; female-individual neurons: *t*_(21)_ = 1.286, *P* = 0.2124). **f3** AP amplitude (male and female combined-individual neurons: *t*_(64)_ = 2.376, *P* = 0.0205; male and female combined-group neurons per mouse: Mann Whitney test, *P* = 0.0152; male-individual neurons: *t*_(41)_ = 2.201, *P* = 0.0334; female-individual neurons: Unpaired t test with Welch’s correction, *P* = 0.0060). **f4** AP rise time (male and female combined-individual neurons: Mann Whitney test, *P* = 0.0080; male and female combined-group neurons per mouse: *t*_(10)_ = 1.900, *P* = 0.0866; male-individual neurons: Mann Whitney test, *P* = 0.0318; female-individual neurons: *t*_(21)_ = 1.900, *P* = 0.0173). **f5** AP half width (male and female combined-individual neurons: Mann Whitney test, *P* = 0.0314; male and female combined-group neurons per mouse: Mann Whitney test, *P* = 0.1017; male-individual neurons: Mann Whitney test, *P* = 0.1743; female-individual neurons: *t*_(21)_ = 3.186, *P* = 0.0044). **f6** AP duration (male and female combined-individual neurons: Mann Whitney test, *P* = 0.0539; male and female combined-group neurons per mouse: Mann Whitney test, *P* = 0.0823; male-individual neurons: Mann Whitney test, *P* = 0.2714; female-individual neurons: *t*_(21)_ = 2.901, *P* = 0.0085). **f7** AP decay time (male and female combined-individual neurons: Mann Whitney test, *P* = 0.0905; male and female combined-group neurons per mouse: *t*_(10)_ = 2.570, *P* = 0.0279; male-individual neurons: Mann Whitney test, *P* = 0.478; female-individual neurons: *t*_(21)_ = 2.712, *P* = 0.0130). **f8** AHP amplitude (male and female combined-individual neurons: *t*_(64)_ = 1.948, *P* = 0.0558; male and female combined-group neurons per mouse: *t*_(10)_ = 0.7414, *P* = 0.4755; male-individual neurons: *t*_(41)_ = 1.504, *P* = 0.1402; female-individual neurons: Mann Whitney test, *P* = 0.6244). **f2–f8** Left, male and female mice combined, individual neurons; middle-left, male and female mice combined, group neurons per mouse; middle-right, male mice-individual neurons; right, female mice-individual neurons. Ctrl: *n* = 35 neurons from three male (19 neurons) and three female (16 neurons) mice. CUS: *n* = 31 neurons from three male (24 neurons) a*n*d three female (7 neurons) mice. *P < 0.05, **P < 0.01, ***P < 0.001 vs control group.

Alterations of intrinsic firing properties of POMC neurons could also contribute to the increased spontaneous firing rates in CUS mice. To explore this possibility, spontaneous, intrinsic action potentials in POMC neurons from *Pomc-GFP* mice were isolated pharmacologically using fast synaptic blockers to inhibit ionotropic glutamate and GABA_A_ receptors. We analyzed the rate, pattern and shape of firing of action potentials of POMC neurons from control mice and mice subjected to 10 days of unpredictable stress (Fig. 3a). The intrinsic firing frequency was increased (Fig. 3b, c) and the membrane potential was depolarized after CUS (Fig. 3d) when data were pooled from males and females. Concomitantly, the percentage of silent POMC neurons (at frequencies <0.5 Hz) decreased from 29% (10 out of 35 neurons recorded) in control mice to 3% (1 out of 31 neurons) in CUS mice. These data indicate that the intrinsic activity of POMC neurons was dramatically increased by CUS. Analysis of the intrinsic firing patterns of POMC neurons revealed a shift in the cumulative frequency distribution of coefficient of variation of interspike intervals to the left and an increase in the firing regularity (Fig. 3e). The intrinsic firing rates correlated negatively with coefficients of variation of interspike intervals under both control and CUS conditions, with a shallower slope in CUS mice (Fig. 3e). Next, we assessed the effects of CUS on action potential waveform parameters (Fig. 3f). CUS had no effect on the threshold (Fig. 3f2) but decreased the amplitude of action potentials (Fig. 3f3). Moreover, POMC neurons from CUS mice exhibited increased action potential rise time (Fig. 3f4) and half-width (Fig. 3f5) and exhibited trends toward greater duration (Fig.  3f6) and decay time (Fig. 3f7). Furthermore, afterhyperpolarization, or AHP, the hyperpolarizing phase of a POMC neuron’s action potential was measured. The amplitude of AHP in POMC neurons was not consistently affected by CUS (Fig. 3f8). These data suggest that chronic exposure to unpredictable stress induces adaptations in the kinetics of action potentials of POMC neurons that may be partially related to changes in intrinsic firing properties.

### Chemogenetic activation of POMC neurons induces anhedonia and behavioral despair

**Fig. 4:**
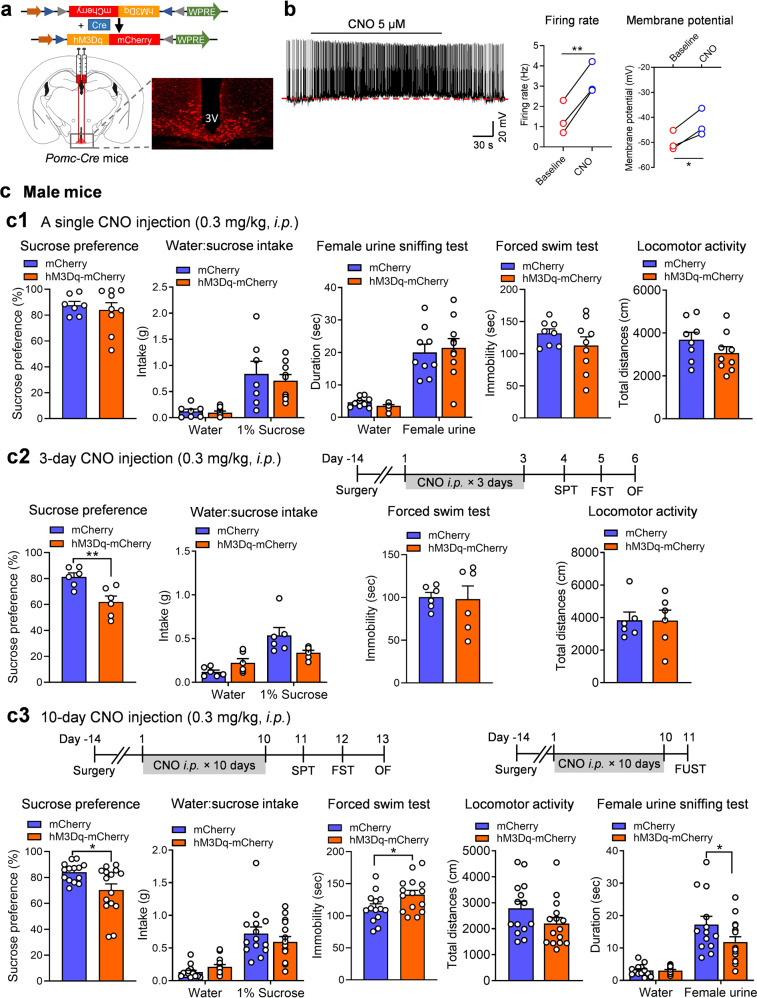

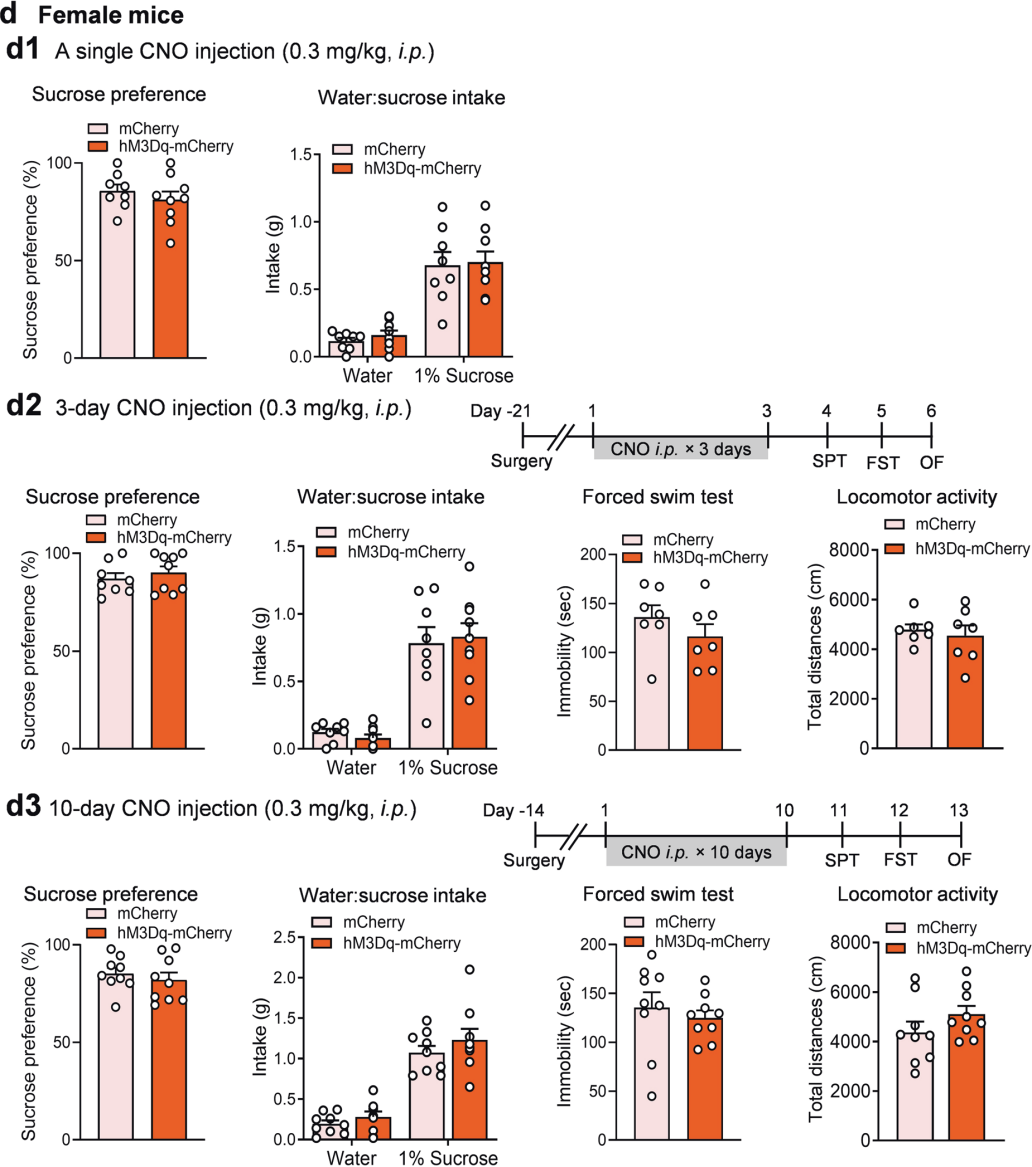
Repeated stimulation of POMC neurons induces behavioral deficits in male mice. **a** Schematic illustration showing stereotaxic injections of AAV-DIO-hM3Dq-mCherry or AAV-DIO-mCherry in the ARC of *Pomc-Cre* mice and a representative image showing mCherry-labeled POMC neurons in the ARC. **b** Left, representative trace of action potentials recorded in POMC neurons expressing hM3Dq in response to bath application of CNO (5 µM); middle, firing rate; right, membrane potential, *n*  =  3 neurons per group. **c** Behavioral responses of male mice to CNO injection (0.3 mg/kg, i.p.). **c1** A single CNO injection. Sucrose preference test (*t*_(14)_ = 0.5232, *P* = 0.6090): AAV-DIO-mCherry, *n* = 7; AAV-DIO-hM3Dq-mCherry, *n*  =  9. Female urine sniffing test (treatment: *F*_(1, 34)_ = 0.006, *P* = 0.9374; odor source: *F*_(1, 34)_ = 72.98, *P* < 0.0001; treatment × odor source: *F*_(1, 34)_ = 0.4274, *P* = 0.5177): AAV-DIO-mCherry, *n*  =  9; AAV-DIO-hM3Dq-mCherry, *n* = 10. Forced swim test (*t*_(15)_ = 1.190, *P* = 0.2525) and locomotor activity (*t*_(15)_ = 1.385, *P* = 0.1863): AAV-DIO-mCherry, *n* = 8; AAV-DIO-hM3Dq-mCherry, *n*  =  9. **c2** Three days of CNO injections (once daily). Sucrose preference, *t*_(10)_ = 3.518, *P* = 0.0056. Forced swim test, unpaired t test with Welch’s correction, *P* = 0.8930. Locomotor activity, *t*_(10)_ = 0.01612, *P* = 0.9875. AAV-DIO-mCherry, *n* = 6; AAV-DIO-hM4Di-mCherry, *n*  =  6. **c3** Te*n* days of CNO injections (once daily). Sucrose preference test (Mann Whitney test, *P* = 0.0292), forced swim test (*t*_(27)_ = 2.211, *P* = 0.0357) and locomotor activity (Mann Whitney test, *P* = 0.0868): AAV-DIO-mCherry, *n*  =  14; AAV-DIO-hM3Dq-mCherry, *n*  =  15. Female urine sniffing test (*F*_(1, 54)_ = 3.410, *P* = 0.0703; odor source: *F*_(1, 54)_ = 60.87, *P* < 0.0001; treatment × odor source: *F*_(1, 54)_ = 3.293, *P* = 0.0751): AAV-DIO-mCherry, *n*  =  13; AAV-DIO-hM3Dq-mCherry, *n*  =  16. **d** Behavioral responses of female mice to CNO injection (0.3 mg/kg, i.p.). **d1** A single CNO injection. Sucrose preference test (*t*_(15)_ = 0.8355, *P* = 0.4165): AAV-DIO-mCherry, *n*  =  8; AAV-DIO-hM3Dq-mCherry, *n*  =  9. **d2** Three days of CNO injections (once daily). Sucrose preference test (Mann Whitney test, P = 0.5403): AAV-DIO-mCherry, *n*  =  8; AAV-DIO-hM3Dq-mCherry, *n*  =  9. Forced swim test (*t*_(12)_ = 1.125, *P* = 0.2827) and locomotor activity (*t*_(12)_ = 0.5193, *P* = 0.6130): *n*  =  7 per group. **d3** Te*n* days of CNO injections (once daily). Sucrose preference test, *t*_(16)_ = 0.6782, *P* = 0.5074. Forced swim test, *t*_(16)_ = 0.6083, *P* = 0.5516. Locomotor activity, *t*_(16)_ = 1.335, *P* = 0.1943. AAV-DIO-mCherry, *n*  =  9; AAV-DIO-hM3Dq-mCherry, *n*  = 9 per group. *P  <  0.05, **P  <  0.01vs mCherry group.

Next, we asked whether acute and chronic activation of POMC neurons can mimic stress-induced behavioral changes. Activation of POMC neurons was achieved by using Cre-dependent, AAV-mediated stimulatory DREADD*-*hM3Dq to depolarize Cre-expressing POMC neurons in *Pomc-Cre* transgenic mice. This method has been widely used to manipulate POMC neuron activity [21, 24]. AAV vectors expressing Cre-dependent hM3Dq, or AAV-DIO-hM3Dq-mCherry, were injected into the ARC of *Pomc-Cre* mice (Fig. 4a). Whole-cell patch clamp electrophysiological recordings confirmed that application of 5 µM CNO to hypothalamic slices increased the firing rates of POMC neurons expressing hM3Dq-mCherrry and depolarized their membrane potential (Fig. 4b). To test whether acute activation of POMC neurons can induce behavioral changes, male *Pomc-Cre* mice received bilateral injections of AAV-DIO-hM3Dq-mCherry or AAV-DIO-mCherry and were injected with a single dose of CNO (0.3 mg/kg, i.p.) 30 min prior to each behavioral test. Sucrose preference was measured within the first 2 h in the dark cycle and showed no difference between the two treatment groups (Fig. 4c1). Sniffing of estrus female urine by male mice is a sex-related reward-seeking behavior [41]. Acute CNO injection failed to produce an effect in the female urine sniffing test in male *Pomc-Cre* mice treated with hM3Dq (Fig. 4c1). These data indicate that acute stimulation of POMC neurons did not affect hedonic responses in male mice. Mice were also tested in the forced swim and locomotor activity tests after an acute CNO injection. Neither behavioral test showed significant differences between hM3Dq- and mCherry-treated male mice (Fig. 4c1). It has been reported that the effects of a single dose of CNO injection can persist more than 9 h [42]. Next, we extended the CNO treatment to 3 days (0.3 mg/kg, once daily) in a separate cohort of male *Pomc-Cre* mice expressing hM3Dq and mCherry. Sucrose preference was significantly decreased by 3 days of activation of POMC neurons; however, immobility in the forced swim test and locomotor activity were unaffected (Fig. 4c2). Next, we asked whether chronic activation of POMC neurons in male mice for 10 days could mimic behavioral deficits induced by CUS. To test this possibility, another cohort of male Pomc-Cre mice expressing hM3Dq and mCherry were treated with CNO (0.3 mg/kg, i.p. once daily) for 10 consecutive days. As shown in Fig. 4c3, 10 days of CNO treatment decreased sucrose preference, increased despair behavior in the forced swim test and induced a trend toward lower locomotor activity in male mice. To test whether chronic stimulation of POMC neurons impacts female urine sniffing time, a separate cohort of male mice were subjected 10 days of CNO injection (0.3 mg/kg daily). Time spent in sniffing female urine was reduced in mice treated with hM3Dq in comparison with those injected with mCherry (Fig. 4c3), suggesting that chronic stimulation of POMC neurons can induce different types of anhedonia in male mice. In contrast to male mice, female mice showed no significant changes in hedonic or despair behaviors following acute (single CNO injection), subacute (3-day CNO injection) or chronic activation (10-day CNO injection) of POMC neurons, as assessed in the sucrose preference test, the forced swim test or the open field test (Fig. 4d). The reason for this difference is unclear, but estrogens in intact, cycling female mice could increase the excitability of POMC neurons [43], which might lead to less responsiveness to CNO-mediated activation. Another possibility could be that female mice are more sensitive to potential confounding effects of anesthesia with ketamine that  has sustained antidepressant properties [44].

### Chemogenetic activation of POMC neurons increases susceptibility to subthreshold levels of unpredictable stress

**Fig. 5:**
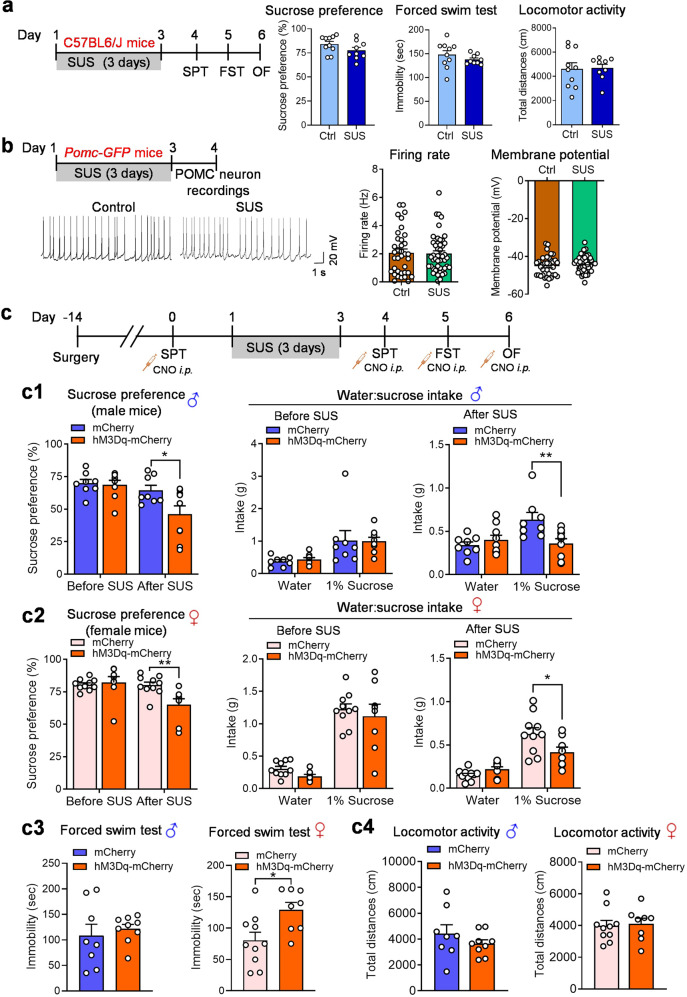
Acute activation of POMC neurons increases susceptibility to subthreshold unpredictable stress (SUS) in both male and female mice. **a** Left, Experimental timeline. Male wild-type C57BL/6J mice (sucrose preference: *t*_(17)_ = 4.578, *P* = 0.1329; forced swim: unpaired *t* test with Welch’s correction, *P* = 0.2852; locomotor activity: *t*_(17)_ = 0.0991, *P* = 0.9222): Ctrl, *n*  =  10; SUS, *n*  =  9. **b** Recordings of POMC neurons from *Pomc-GFP* mice after exposure to SUS. Left-top, experimental timeline; left-bottom, representative whole-cell recording traces of POMC neurons; middle, firing rates (Mann Whitney test, *P* = 0.8874); right, membrane potential (*t*_(81)_ = 1.248, *P* = 0.2157). Ctrl: *n*  =  36 neurons from two male (15 neurons) and two female (21 neurons) mice. SUS: *n*  =  47 neurons from two male (22 neurons) and two female (25 neurons) mice. **c** A combination of stimulation of POMC neurons and SUS exposure in *Pomc-Cre* mice. Upper panel, experimental timeline. **c1** Sucrose preference test in male mice (before SUS: Mann Whitney test, *P* = 0.6930; after SUS: *t*_(15)_ = 2.391, *P* = 0.0304). **c2** Sucrose preference test in female mice (before SUS: Mann Whitney test: *P* = 0.1728; after SUS: Mann Whitney test: *P* = 0.0062). **c3** Forced swim test in male (left, unpaired t test with Welch’s correction, *P* = 0.5930) and female (right, *t*_(16)_ = 2.703, *P* = 0.0157) mice. **c4** Locomotor activity of male (left, unpaired *t* test with Welch’s correction, *P* = 0.3261) and female (right, *t*_(16)_ = 0.2257, *P* = 0.8243) mice. Male: AAV-DIO-mCherry, *n*  =  8; AAV-DIO-hM3Dq-mCherry, *n*  =  9. Female: AAV-DIO-mCherry, *n*  =  10; AAV-DIO-hM3Dq-mCherry, *n*  = 8. *P < 0.05, **P < 0.01 vs control or mCherry group.

Our next question was whether acute activation of POMC neurons could increase susceptibility to subthreshold levels of unpredictable stress. We have previously shown that mice exposed to 3 days of unpredictable stress show no significant change in sucrose preference [1]. In the present study, multiple behavioral tests, including sucrose preference, forced swimming and open field tests, were conducted to assess behavioral consequences 1 day after exposure to unpredictable stress (first 3 days in Table 1). As expected, none of these behaviors were significantly altered by this short duration of unpredictable stress (Fig. 5a). Thus, this stress paradigm was used as a subthreshold form of unpredictable stress (SUS) to assess the impact of selective activation of POMC neurons on stress susceptibility. We have previously shown that POMC neurons can be rapidly activated by acute stress, as evidenced by c-fos induction [4]. Given the findings described above that 10 days of CUS increased the spontaneous firing activity of POMC neurons, we first asked whether the SUS protocol can induce long-lasting changes in neuronal firing activity of POMC neurons. To address this question, mice were subjected to 3 days of SUS (Table 1) and POMC neurons were recorded in hypothalamic slices 1 day after the last stress exposure. We found that the firing rate and the membrane potential of POMC neurons were not significantly affected by SUS (Fig. 5b). To test whether acute activation of POMC neurons increases susceptibility to stress, mice expressing hM3Dq and mCherry in *Pomc-Cre* neurons were tested for sucrose preference after acute CNO injection, then subjected to 3 days of SUS followed by behavioral tests after acute CNO injection (Fig. 5c). As shown in Fig. 4c1, d1, acute activation of POMC neurons by a CNO injection had no effect on sucrose preference in male or female mice prior to SUS exposure but significantly reduced sucrose preference in both male and female mice after SUS exposure (Fig. 5c1, c2) and increased immobility time in the forced swim test in female but not male mice (Fig. 5c3). Neither male nor female mice showed significant changes in locomotor activity (Fig. 5c4), which suggests that the forced swim results were not confounded by non-specific changes in mobility. These results indicate that acute activation of POMC neurons increases stress susceptibility in both male and female mice.

### Chemogenetic inhibition of POMC neurons is sufficient to reverse anhedonia and despair behavior induced by CUS

**Fig. 6:**
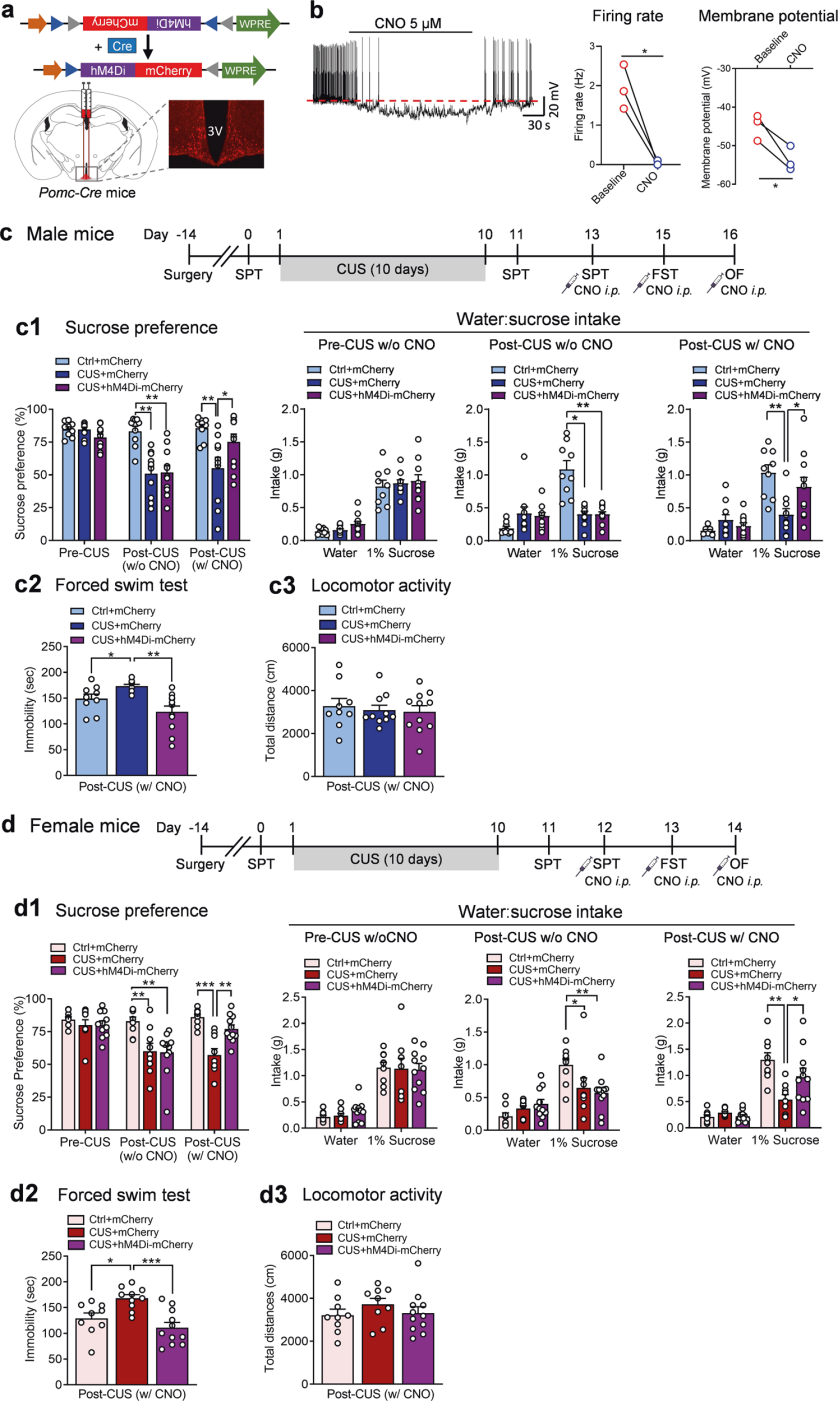
Inhibition of POMC neurons reverses chronic unpredictable stress-induced behavioral deficits. **a** Schematic illustration showing stereotaxic injection of AAV-DIO-hM4Di-mCherry or AAV-DIO-mCherry in the ARC of *Pomc-*Cre mice and a representative image showing mCherry-labeled POMC neurons. **b** Left, representative traces of action potentials recorded in POMC neurons expressing hM4Di in response to bath application of CNO (5 µM); middle, firing rate (paired t-test, *t*_(2)_ = 5.756, *P* = 0.0289); right, membrane potential (paired t-test, *t*_(2)_ = 4.488, *P* = 0.0462). *n* = 3 neurons per group. **c** Timeline of experimental procedures in male *Pomc-*Cre mice. **c1** Sucrose preference test before and after CUS without or with acute CNO injection (0.3 mg/kg, i.p.). Pre-CUS: Kruskal-Wallis test, *P* = 0.1529; post-CUS w/o CNO: Kruskal-Wallis test, *P* < 0.001; post-CUS w/ CNO: Kruskal-Wallis test, *P* = 0.0143. **c2** Forced swim test after CUS with acute CNO injection (Brown-Forsythe ANOVA test, *P* = 0.0015). **c3** Locomotor activity after CUS with acute CNO injection (*F*_(2,27)_ = 0.2077, *P* = 0.8138). Ctrl+mCherry, *n* = 9; CUS + mCherry, *n* = 10; CUS + hM4Di-mCherry, *n* = 11. **d** Timeline of experimental procedures in female *Pomc-*Cre mice. **d1** Sucrose preference test before and after CUS without or with acute CNO injection. Pre-CUS: Kruskal-Wallis test, *P* = 0.6505; post-CUS w/o CNO: Kruskal-Wallis test, *P* = 0.0023; post-CUS w/CNO: *F*_(2,26)_  =  16.77, *P* < 0.001. Ctrl+mCherry, *n*  =  9; CUS + mCherry, *n*  =  9; CUS + hM4Di-mCherry, *n*  =  11. **d2** Forced swim test after CUS with acute CNO injection (*F*_(2,27)_  =  10.05, *P* < 0.001). Ctrl+mCherry, *n*  =  9; CUS + mCherry, *n*  =  10; CUS + hM4Di-mCherry, *n*  =  11. **d3** Locomotor activity after CUS with acute CNO injection (*F*_(2,26)_  =  0.8191, *P* = 0.4519). Ctrl+mCherry, *n*  =  9; CUS + mCherry, *n*  =  9; CUS + hM4Di-mCherry, *n*  =  11. *P < 0.05, **P < 0.01, ***P < 0.001 vs Ctrl+mCherry group or CUS + mCherry group.

We next asked whether inhibition of POMC neurons can reverse CUS-induced behavioral deficits. First, we confirmed the effect of CNO on POMC neurons by whole-cell patch clamp recordings from hM4Di-expressing POMC neurons from *Pomc-Cre* mice injected with AAV-DIO-hM4Di-mCherry in the ARC (Fig. 6a). CNO application decreased the firing rate and hyperpolarized the membrane potential (Fig. 6b), demonstrating that CNO-mediated activation of hM4Di inhibited the activity of POMC neurons. To test the behavioral effects of CNO-induced inhibition of POMC neurons, male and female *Pomc-Cre* mice received intra-ARC injection with AAV-DIO-hM4Di-mCherry or AAV-DIO-mCherry and were then divided into two groups for 10 days of CUS exposure or daily brief handling as control. The hM4Di- and mCherry-treated mice showed no differences in their sucrose preference prior to stress exposure and in the absence of CNO injection (Fig. 6c1, d1). As shown previously [1], CUS significantly decreased sucrose preference in both male and female mice prior to CNO injection (Fig. 6c1, d1). This reduction was reversed by an acute CNO injection (0.3 mg/kg, i.p.) in *Pomc-Cre* mice expressing hM4Di, compared with mCherry-expressing control *Pomc-Cre* mice (Fig. 6c1, d1). In addition, CUS induced despair behavior, as indicated by increased immobility in the forced swim test; this effect was also reversed by acute inhibition of POMC neurons with CNO injection in hM4Di-expressing *Pomc-Cre* mice (Fig. 6c2, d2), whereas locomotor activity was not altered by either CUS or CNO treatment (Fig. 6c3, d3). These results suggest that acute inhibition of POMC neurons is sufficient to reverse CUS-induced behavioral deficits.

## Discussion

In the ARC of the hypothalamus, POMC and AgRP neurons are well-positioned to relay and integrate peripheral and central signals to elicit adaptive and maladaptive behavioral responses to environmental challenges. In parallel with the investigations of synaptic and intrinsic plasticity of AgRP neurons using a CUS paradigm [1], this study assessed the impact of the same chronic stress paradigm on POMC neuron firing and behavioral consequences of DREADD-mediated control of POMC neuron activity. We demonstrated that CUS depolarized POMC neurons and increased their firing rates through modulating both synaptic and intrinsic neuronal properties. Repeated activation of POMC neurons was sufficient to induce anhedonia and behavioral despair, mimicking repeated exposure to stress. By contrast, acute inhibition of POMC neurons was able to reverse behavioral deficits induced by CUS. Collectively, these data suggest that POMC neurons are both necessary and sufficient for chronic stress-induced behavioral phenotypes.

Anhedonia, loss of interest and pleasure, is a common symptom in depression and other psychiatric illnesses. The ARC has been shown to be involved in reward processing and motivated behaviors [45, 46], but only recently dysfunction of specific neuronal populations in the ARC was discovered to be associated with stress-induced anhedonia [1, 9]. Using the same CUS paradigm, we have demonstrated that chronic stress hyperpolarizes AgRP neurons and decreases their firing rates [1].  Furthermore, inhibition of AgRP neurons increases stress susceptibility, whereas activation of AgRP neurons reverses anhedonia and behavior despair in CUS mice [1]. In contrast to the impact of CUS on AgRP neurons, we found that CUS depolarizes POMC neurons and increases their firing frequency. Notably, in the present study, the whole-cell patch clamp recordings of POMC and AgRP neurons were performed 1 day after the final stress session to eliminate acute stress effects. This is in contrast to a recent study that recorded the activity of POMC and AgRP neurons immediately after exposure to restraint stress [9]. Given that POMC neurons have been shown to be activated quickly by restraint stress, as evidenced by c-fos induction 30 min after stress exposure [4], it is not surprising that POMC neuron firing was increased in mice recorded after a single exposure to restraint stress or immediately following the last stress session of repeated restraint stress [9]. The initial activation of neurons in response to acute stress has been reported to be followed by a decline or depression of neuronal activity with the termination of stress [47]. Indeed, when recorded at 1 day following restraint stress in a subthreshold unpredictable stress paradigm (3 days of stress exposure), we observed no change in POMC neuron firing. These findings suggest that chronicity, unpredictability and variability in stress exposure are important factors in driving persistent hyperactivity of POMC neurons.

POMC neurons in the ARC receive both GABAergic and glutamatergic inputs from multiple brain regions [14, 48, 49]. The observed hyperactivity of POMC neurons following chronic stress exposure could result from modulation of excitatory and inhibitory synaptic transmission [50–53]. Under basal conditions, there are more excitatory than inhibitory synapses on POMC neurons [49]. We found that CUS had no effect on excitatory synaptic transmission, but decreased inhibitory synaptic inputs to POMC neurons. The frequency of spontaneous IPSCs was decreased in POMC neurons following CUS, reflecting presynaptic modifications. Furthermore, this decrease was eliminated by blocking action potential formation and its propagation, suggesting that CUS induces a presynaptic hyperpolarization in GABAergic terminals which synapse onto POMC neurons. Given that AgRP neurons can release GABA onto POMC neurons in the ARC [54] and that AgRP neurons are hyperpolarized by CUS [1], it is reasonable to assume that the decreased inhibitory synaptic transmission in POMC neurons is caused in part by hyperpolarized GABAergic AgRP terminals. This notion is supported by the findings that ablation of AgRP neurons causes a dramatic reduction in spontaneous GABAergic synaptic transmission in POMC neurons [55], and that optogenetic stimulation of AgRP neurons inhibits POMC neuron firing [54, 56]. However, some studies reported that AgRP neurons may not be a primary source of GABA onto POMC neurons, and the relevance of GABAergic inputs from AgRP to POMC neurons is state-dependent [48, 50, 57, 58]. The exact interplay between POMC neurons and AgRP neurons in stress responses and adaptations requires further investigation. Nonetheless, CUS-induced weakening of GABAergic inputs to POMC neurons, in the absence of changes in glutamatergic inputs, would cause the synaptic excitation/synaptic inhibition balance to shift toward excitation. This could contribute to hyperactivity of POMC neurons.

In POMC neurons, persistent increases in intrinsic excitability occur in parallel with synaptic modifications following CUS. The intrinsic neuronal excitability determines the translation of synaptic input to the output function of a given neuron. One possible mechanism for increased intrinsic excitability of POMC neurons is the regulation of expression and distribution of ion channels inserted into the membrane of POMC neurons that contribute to the electrical properties and depolarization potential [59, 60]. POMC neurons were reported to possess ATP-sensitive potassium (K_ATP_) channels and express the K_ATP_ channel subunits Kir6.2 and SUR1 [61, 62]. K_ATP_ channel openers induce an outward K^+^ current in the vast majority of POMC neurons [62], leading to membrane hyperpolarization and reduced neuronal activity [62, 63]. Conversely, pharmacological blockade of K_ATP_ channels can activate POMC neurons [61]. These studies suggest the importance of K_ATP_ channels for normal activity of POMC neurons. However, it is unknown whether CUS suppresses expression and/or function of the K_ATP_ channels, leading to closure of the channels. Alternatively, inhibition of Ca^2+^-dependent K^+^ (SK) currents may contribute to hyperactivity of POMC neurons observed in this study. Blocking SK channels was reported to reactivate POMC neurons [60]. Future investigations of ion channel regulation will provide insights into the mechanisms underlying CUS-induced hyperactivity of POMC neurons.

Notably, POMC neurons exhibited higher degrees of firing regularity after CUS exposure, while they fire spontaneously in an irregular manner under control conditions. The mechanisms driving variability in spike-timing of POMC neurons are unknown. Dendritic morphology plays a critical role in determining neuronal firing patterns [64–67]. Chronic stress alters dendritic morphology in many brain regions [68, 69]. It is possible that CUS may induce changes in dendritic morphology of POMC neurons, contributing to firing regularity. Another determinant of neuronal firing patterns is the composition and density of ion channels [67, 70]. It has been shown that SK channels control firing regularity by modulating sodium channel availability [71, 72]. Voltage-gated K channels [73–75] and HCN channels [76, 77] are also involved in regulating the waveform and spike regularity. In addition, the firing regularity can be influenced by the properties and variability of synaptic inputs [78, 79]. Future studies will identify the key mechanism that controls firing patterns of POMC neurons and how firing regularity influences neuronal information processing.

While exposure to CUS induced similar effects on POMC neuron excitability in male and female mice, behavioral responses to repeated activation of POMC neurons exhibited sex differences. Repeated activation of POMC neurons in stress-naïve male mice for 3 or 10 days induced behavioral deficits, including decreased sucrose preference, decreased sex-related reward seeking behavior and increased behavioral despair. However, stress-naïve female mice failed to show any behavioral changes. Although acute activation of POMC neurons was not sufficient to induce significant behavioral effects in stress-naive mice, the susceptibility of both male and female mice to subthreshold unpredictable stress was increased by acute stimulating POMC neurons. On the other hand, acute inhibition of POMC neurons was able to rescue behavioral deficits induced by CUS in both male and female mice. These studies suggest that hyperactivity of POMC neurons is required for the induction and expression of CUS-induced behavioral deficits. Among behavioral tests, sucrose preference has been widely used as a reliable measure of anhedonia in both male and female mice [80]. It is conceivable that the impact of altering POMC neuron activity on sucrose preference could be consequential to changes in caloric consumption rather than a true preference for sweet taste. In this study, however, sucrose preference was conducted within the first 2 h in the dark cycle in mice provided with free access to food and water. Previous studies have shown that neither chemogenetic activation by i.p. injection or continuous infusion of CNO, nor optogenetic activation of POMC neurons, affects food intake within 2 h [17, 21, 24, 25]. Thus, the observed changes in sucrose preference were unlikely to reflect the impact of POMC neuron activity on metabolism.

Collectively, our findings indicate that activation of POMC neurons in the ARC is both necessary and sufficient to mediate stress susceptibility and induce anhedonia and behavioral despair. Further studies investigating the mechanisms underlying the synaptic disinhibition and intrinsic hyperexcitability of POMC neurons will provide insight into how POMC neurons modulate stress-related behaviors. Together with our previous findings that stimulating AgRP neurons decreases stress susceptibility and reverses CUS-induced behavioral deficits [1], these results suggest that POMC neurons act in opposition to AgRP neurons in behavioral and neural plasticity to chronic stress. Thus, hypothalamic POMC and AgRP neurons can be viewed as Yin-Yang partners in modulating responses and adaptations to stress. Whether efferent projections from these neurons converge on the same downstream targets to control behavioral susceptibility to stress, and whether their influences require melanocortin receptor signaling, need to be investigated.

## Supplementary information

Supplementary Information
