# Supplementary material for: Increased intrinsic and synaptic excitability of hypothalamic POMC neurons underlies chronic stress-induced behavioral deficits
Source: Mol Psychiatry. 2022 Dec 6;28(3):1365–82. doi: 10.1038/s41380-022-01872-5 (PMC10005948; doi:10.1038/s41380-022-01872-5)
Supplement: Supplementary file 1 — Supplementary Information [file 41380_2022_1872_MOESM1_ESM.docx]

**Supplementary Information**

**Materials and Methods**

**Animals**

Male *Pomc-Cre* mice were crossed with Ai14 tdTomato female mice to obtain *Pomc-Cre;tdTomato* mice with tdTomato fluorescence in Cre-expressing cells, which was used to identify POMC neurons. The offspring were genotyped by PCR amplification of tail DNA. The Gt(ROSA)26Sor^tm14(CAG-tdTomato)^ allele and *Pomc*-*Cre* allele were determined using the primers 5’-GGC ATT AAA GCA GCG TAT CC-3’ and 5’-CTG TTC CTG TAC GGC ATG G-3’ and the primers 5’-GCG GTC TGG CAG TAA AAA CTA TC-3’and 5’-GTG AAA CAG CAT TGC TGT CAC TT-3’, respectively.

Animals were housed in groups of 3-5 under a 12/12-h light/dark cycle (lights on at 0600 h) with *ad libitum* access to water and standard food pellets. All animal procedures were approved by the Institutional Animal Care and Use Committees of University of Texas Health Science Center at San Antonio and Augusta University.

**Viral injections**

*Pomc-Cre* mice at 7 weeks of age were anesthetized with an intraperitoneal injection of a cocktail containing xylazine (10 mg per kg) and ketamine (100 mg per kg) diluted in saline and mounted onto a stereotaxic frame, as described elsewhere^1-3^. Cre-dependent AAV5-hSyn-DIO-hM3D(Gq)-mCherry (referred to as AAV-DIO-hM3Dq-mCherry; titer 8.2 × 10^12^ viral genomes per ml), AAV5-hSyn-DIO-hM4D(Gi)-mCherry (referred to as AAV-DIO-hM4Di-mCherry; titer 5.3 × 10^12^ viral genomes per ml) and AAV5-hSyn-DIO-mCherry (referred to as AAV-DIO-mCherry; titer 1.8 × 10^12^ viral genomes per ml) were gifts from Bryan Roth (Addgene Viral Prep #44361-AAV5, #50475-AAV5 and #50459-AAV5, respectively) ^4^. A volume of 0.2 μL AAV vectors was delivered bilaterally into the ARC (coordinates: AP -1.4 mm, ML ± 0.2 mm, DV -5.8 mm from bregma) at a rate of 0.1 μL/min with a 33-gauge stainless steel injector connected to a UMP3 microsyringe pump (World Precision Instruments, Sarasota, FL). Additional 5 min were allowed for diffusion and prevention of backflow. Behavioral procedures were conducted 14 days post-AAV injection. The injection sites were verified by examining mCherry fluorescence in each animal at the end of the experiments. Animals with missed injections were excluded from statistical analysis.

**Whole-cell patch-clamp recordings**

Electrophysiological recordings were performed as previously described ^3, 5^. Mice were anesthetized with isoflurane and brains were quickly removed and transferred to an ice-cold solution (254 mM sucrose, 3 mM KCl, 2 mM MgCl_2_, 2 mM CaCl_2_, 1.25 mM NaH_2_PO_4_, 10 mM D-glucose, and 24 mM NaHCO_3_). A tissue block containing the hypothalamus was immediately dissected. Coronal brain slices (approx. 300 μm) were prepared with a Leica VT1000S Vibratome (Leica Microsystems), recovered at 32 °C for 30 min and subsequently at room temperature for another 30 min in an oxygenated (95% O_2_/5% CO_2_) artificial cerebrospinal fluid solution (aCSF, 124 mM NaCl, 2 mM KCl, 2 mM MgSO_4_, 2 mM CaCl_2_, 1.25 mM NaH_2_PO_4_, 26 mM NaHCO_3_, and 10 mM Glucose, with pH 7.3 and osmolarity 300 mOsm/L) prior to recordings. Slices were transferred to the recording chamber and superfused with oxygenated aCSF at a flow rate of 2 ml/min at room temperature.

Neurons were visualized with a fixed stage upright microscope (Examiner.A1, Zeiss, NY) using 5× and 40× water-immersion objectives with infrared differential interference contrast optics (IR-DIC) and fluorescence optics. POMC neurons were identified by their anatomical location in the ARC of the hypothalamus and by their fluorescence, which was visualized with Calibri.2 illumination system (Zeiss) in combination with a filter. Patch electrodes with a final resistance of 3–5 MΩ were prepared by a P-97 micropipette puller (Sutter Instrument, CA). For current-clamp recording and excitatory postsynaptic currents (EPSCs) recording, pipettes were filled a potassium gluconate-based internal solution (120 mM potassium gluconate, 20 mM KCl, 2 mM MgCl_2_, 10 mM HEPES, 2 mM ATP, 0.25 mM GTP and 0.1 mM EGTA adjusted to pH 7.4 and osmolarity of 295 mOsm/L). For inhibitory postsynaptic currents (IPSCs) recording, pipettes were filled with a CsCl-based internal solution (140 mM CsCl, 5 mM MgCl_2_, 10 mM HEPES, 2 mM ATP, 0.3 mM GTP and 1 mM BAPTA, adjusted to pH 7,4 and osmolarity of 295 mOsm/L). All recordings were made using a MultiClamp 700B Microelectrode Amplifier (Molecular Devices, LLC., CA), and data was filtered at 2 kHz and digitized at 10 kHz by using Axon Digidata 1550A (Axon Instruments) and analyzed on a PC computer with pCLAMP 10.7 program (Molecular Devices, LLC., CA).  The series resistance was measured before patch clamp recording, and only those cells with series resistance less than 20 MΩ were recorded. After the recording was completed, the series resistance was measured again, and the cells were excluded for further analysis if the series resistance varied by more than 15%. Membrane potential and spontaneous action potential (AP) firing rates were measured by whole-cell patch-clamp recording in current-clamp mode in the absence and presence of fast synaptic blockers, using 100 μM picrotoxin, 10 μM 6-cyano-7-nitroquinoxaline-2,3-dione (CNQX), and 50 μM DL-2-amino-5-phosphonopentanoic acid (DL-AP5) to inhibit GABA_A_ and ionotropic glutamate receptors AMPAR and NMDAR in order to isolate spontaneous, intrinsic action potentials. The interspike intervals (ISIs) for each neuron were measured; coefficient of variations (the ratio of the standard deviation of ISI to the mean of ISI) were calculated. The resting membrane potential was used as the baseline value for the measurements of the AP amplitude and afterhyperpolarization amplitude. AP duration, rise time, decay time, and half-width were determined using Clampfit 10.7 software (Axon Instruments). AP waveform was signal-averaged over three to five firing cycles for each neuron ^6^.

EPSCs and IPSCs were recorded from identified POMC neurons under voltage-clamp mode with membrane potential held at -60 mV. Specifically, spontaneous EPSCs were recorded in the presence of 100 μM picrotoxin to eliminate ionotropic GABAergic transmission. Recordings of spontaneous IPSCs were made in the presence of AMPA and NMDA receptor antagonists (10 µM CNQX and 50 µM DL-AP5) to block glutamatergic responses. Miniature IPSCs and miniature EPSCs were recorded with 1 μM tetrodotoxin (TTX) to block sodium channels, preventing action potential generation and propagation. To analyze synaptic events, template search was performed offline by Clampfit (Molecular Devices, LLC., CA). Inter-event interval and peak amplitude of each synaptic event were measured. The average amplitude and frequency of synaptic events were calculated at the end of analysis for each neuron.

**Behavioral procedures**

***Sucrose preference test.*** Mice were habituated to drinking from two bottles for 1 week before testing. To measure the preference for sucrose solution, the animals were tested with a free choice of one bottle of water and one bottle of 1% sucrose solution during the first two hours of the dark cycle. Water and sucrose intake were measured, and the preference for sucrose was calculated by dividing the weight of sucrose intake by the total weight of fluid intake.

***Female urine sniffing test.*** This non-operant test was used to assess sex-related reward-seeking behavior based upon interest of male rodents in pheromonal odors from estrus female urine ^7^. Male mice were subjected to the following test procedure: (1) 3-min exposure to the cotton tip dipped in water; (2) a 45-min interval; (3) 3-min exposure to the cotton tip dipped in fresh urine collected from female mice in the estrus phase. The duration of female urine sniffing time was scored.

***Forced swim test.*** Mice were placed in a clear Plexiglas cylinder (25 cm high; 10 cm in diameter) filled with 24 °C water to a depth of 15 cm. A camera positioned directly above the cylinder was used to record the behavior of each mouse for 6 min. The duration of immobility in the last 4 min was measured. Immobility was defined as no movement of the limb or body except those caused by respiration ^8-10^.

***Locomotor activity.*** Mice were placed in the SuperFlex Fusion open field cage (40 × 40 × 30 cm^3^) containing a 16 × 16 photobeam array for the x/y-axis and 16 photobeams for the z-axis (Omnitech Electronics Inc., OH) and allowed to freely explore for 30 min under dim illumination conditions. The movements of mice were monitored by infrared photosensors equipped on the cage, and the total distance traveled was analyzed using Fusion software (Omnitech Electronics Inc., OH).

**Statistical analysis**

For normally distributed data, two-tailed t tests were used to assess differences between two experimental groups with equal variances. For a two-sample comparison of means with unequal variances, two-tailed t tests with the Welch’s correction were used. Two-tailed, paired t tests were used to compare membrane potential and firing rates before and after CNO incubation. One-way analyses of variance (ANOVAs) followed by Bonferroni *post hoc* tests were used for comparing three or more groups. For non-normally distributed data, Mann-Whitney U tests were performed to compare two groups. For analysis of three or more groups with non-normal distribution, the Kruskal-Wallis test followed by Dunn's multiple comparisons was used. For comparison of three or more groups with unequal variances, the Brown-Forsythe test and Welch ANOVA test were applied followed by Dunnett's T3 multiple comparisons. Two-way ANOVAs followed by Bonferroni post hoc tests were used for analysis of the female urine sniffing test results. Cumulative probabilities of amplitude and inter-event interval distributions of postsynaptic currents were analyzed with the two-tailed Kolmogorov–Smirnov test. For correlation analysis, Pearson correlation coefficients or nonparametric Spearman correlation coefficients were computed for normally or non-normally distributed data, respectively. *P* < 0.05 was considered statistically significant.

**References**

1. Fang X, Jiang S, Wang J, Bai Y, Kim CS, Blake D *et al.* Chronic unpredictable stress induces depression-related behaviors by suppressing AgRP neuron activity. *Mol Psychiatry* 2021; **26**(6)**:** 2299-2315.

2. Sun FJ, Lei Y, You JJ, Li C, Sun LS, Garza J *et al.* Adiponectin modulates ventral tegmental area dopamine neuron activity and anxiety-related behavior through AdipoR1. *Mol Psychiatr* 2019; **24**(1)**:** 126-144.

3. Lei Y, Wang J, Wang D, Li C, Liu B, Fang X *et al.* SIRT1 in forebrain excitatory neurons produces sexually dimorphic effects on depression-related behaviors and modulates neuronal excitability and synaptic transmission in the medial prefrontal cortex. *Mol Psychiatry* 2020; **25**(5)**:** 1094-1111.

4. Krashes MJ, Koda S, Ye C, Rogan SC, Adams AC, Cusher DS *et al.* Rapid, reversible activation of AgRP neurons drives feeding behavior in mice. *J Clin Invest* 2011; **121**(4)**:** 1424-1428.

5. Zhang D, Wang X, Wang B, Garza JC, Fang X, Wang J *et al.* Adiponectin regulates contextual fear extinction and intrinsic excitability of dentate gyrus granule neurons through AdipoR2 receptors. *Mol Psychiatr* 2017; **22**(7)**:** 1044-1055.

6. Kimm T, Khaliq ZM, Bean BP. Differential Regulation of Action Potential Shape and Burst-Frequency Firing by BK and Kv2 Channels in Substantia Nigra Dopaminergic Neurons. *J Neurosci* 2015; **35**(50)**:** 16404-16417.

7. Malkesman O, Scattoni ML, Paredes D, Tragon T, Pearson B, Shaltiel G *et al.* The female urine sniffing test: a novel approach for assessing reward-seeking behavior in rodents. *Biol Psychiatry* 2010; **67**(9)**:** 864-871.

8. Liu J, Perez SM, Zhang W, Lodge DJ, Lu XY. Selective deletion of the leptin receptor in dopamine neurons produces anxiogenic-like behavior and increases dopaminergic activity in amygdala. *Mol Psychiatr* 2011; **16**(10)**:** 1024-1038.

9. Liu J, Guo M, Zhang D, Cheng SY, Liu ML, Ding J *et al.* Adiponectin is critical in determining susceptibility to depressive behaviors and has antidepressant-like activity. *P Natl Acad Sci USA* 2012; **109**(30)**:** 12248-12253.

10. Guo M, Li C, Lei Y, Xu S, Zhao D, Lu XY. Role of the adipose PPARgamma-adiponectin axis in susceptibility to stress and depression/anxiety-related behaviors. *Molecular psychiatry* 2017; **22**(7)**:** 1056-1068.
